# Nutritional impact of mycotoxins in food animal production and strategies for mitigation

**DOI:** 10.1186/s40104-022-00714-2

**Published:** 2022-06-08

**Authors:** Ran Xu, Elijah G. Kiarie, Alexandros Yiannikouris, Lvhui Sun, Niel A. Karrow

**Affiliations:** 1 Department of Animal Biosciences, University of Guelph, Guelph, ON N1G 2W1 Canada; 2 Alltech Inc., Center for Animal Nutrigenomics and Applied Animal Nutrition, 3031 Catnip Hill, Road, Nicholasville, KY 40356 USA; 3 Hubei Hongshan Laboratory, College of Animal Science and Technology, Huazhong Agricultural University, Wuhan, 430070 Hubei China

**Keywords:** Food animals, Mitigation strategies, Mycotoxins, Nutritional impact, Risk management, Susceptibility differences

## Abstract

Mycotoxins are toxic secondary metabolites produced by filamentous fungi that are commonly detected as natural contaminants in agricultural commodities worldwide. Mycotoxin exposure can lead to mycotoxicosis in both animals and humans when found in animal feeds and food products, and at lower concentrations can affect animal performance by disrupting nutrient digestion, absorption, metabolism, and animal physiology. Thus, mycotoxin contamination of animal feeds represents a significant issue to the livestock industry and is a health threat to food animals. Since prevention of mycotoxin formation is difficult to undertake to avoid contamination, mitigation strategies are needed. This review explores how the mycotoxins aflatoxins, deoxynivalenol, zearalenone, fumonisins and ochratoxin A impose nutritional and metabolic effects on food animals and summarizes mitigation strategies to reduce the risk of mycotoxicity.

## Introduction

A rapidly growing world population and increased standard of living in many countries has driven increasing demand for high quality food. The livestock industry contributes to global food supply and human society by converting animal feed, which is generally made from low-value agricultural products or by-products to high value animal-derived food products that are rich in nutrients such as meat, eggs, and milk. For food animals to achieve their genetically selected production potential, nutrients must be made available and partitioned towards productive functions. The overall availability of these resources is determined by food intake, gastrointestinal digestion, and absorption [1], thus limiting factors of nutrient availability could constrain animal performance.

Mycotoxins are secondary metabolites produced by filamentous fungi as coping strategies under environmental pressure and functioning as fitness factors to enhance pathogenicity, aggressiveness and/or virulence the virulence of fungi [2, 3]. Mycotoxins are among one of the most significant hazards to the feed supply chain and pose a threat to feed industries worldwide with a direct impact on feed safety, animal health and productivity, human health via animal by-products, economies and international trade [4–6]. These toxic compounds are commonly detected as natural contaminants in a variety of agricultural commodities of plant origin, especially in cereal grains, and are therefore often detected in animal feeds containing corn, soybean, and wheat [5, 7–9], but can also be present in silage, haylage and pasture [10–12]. Major mycotoxins can also be found in animal-derived products such as eggs, meat, milk and milk by-products with varying residual concentrations due to carry-over from animals that have consumed contaminated feeds [13, 14]. Natural co-occurrence of mycotoxins with additive, antagonistic, or synergistic is more common in foods and feeds than single mycotoxin contaminants [15, 16]. The ingestion of mycotoxin-contaminated feeds by animals can cause both acute and chronic toxicity. However, a major problem associated with mycotoxin contamination in livestock production is the chronic effects on growth, production and immune function associated with a range of metabolic, physiological, and immunological disturbances in animals induced by the ingestion of low levels of a single or combined mycotoxins, which could result in economic losses [4, 17]. This review specifically focuses on the nutritional effects of mycotoxins on food animals. The mycotoxins most commonly addressed in this review include aflatoxins (AF), deoxynivalenol (DON), zearalenone (ZEN), fumonisins (FUM) and ochratoxin A (OTA) based on their prevalence, toxicity and relevance to food animals. In addition, the main mitigation strategies for mycotoxin contamination are presented in this review.

## Major mycotoxins

Aflatoxins are produced pre- and post-harvest, mainly by *Aspergillus flavus* and *A. parasiticus* [7]. Among the AF family, AFB_1_ is the most commonly occurring and is considered to be the most potent carcinogenic toxin known to both animals and humans [5]. A wide variety of agricultural commodities including corn, wheat, and rice can be contaminated with AF [7]. Two common climate conditions associated with tropical and subtropical regions favor AF contamination of crops; high temperature and low humidity facilitate pre-harvest production and accumulation of AF in the growing plants [18, 19], on the other hand, exposure to high temperatures and high moisture leading up the harvest and during crop storage also favors fungal growth and AF production [20].

The trichothecenes (TRC) are produced predominately by the *Fusarium* species [5], which usually infect crops and produce mycotoxins in the field, generally in association with a cool and excessively wet conditions [8]. Deoxynivalenol is produced mainly by *F. graminearum* and *F. culmorum* and is the predominate TRC considering it is the most widely distributed and the most frequently detected mycotoxin in cereal grains such as corn, wheat, barley, and oats worldwide. Exposure to DON has been reported to have negative impacts on animals, especially pigs, which makes it a highly relevant mycotoxin to livestock husbandry even though it is among the least acutely toxic TRC in the family [21]. DON can interact with the neural dopaminergic system after ingestion and cause nausea [22]. It can also inhibit protein synthesis [23].

Zearalenone is another major mycotoxin primarily produced by *F. graminearum*, but can also be produced by *F. culmorum*, *F. cerealis* and *F. equiseti*. Zearalenone contaminates cereals worldwide [21].

The FUM are produced by several species of *Fusarium* among which *F. verticillioides* and *F. proliferatum* are the main producers [21]. High temperatures and low precipitation around corn silking facilitate fungal infection and subsequent FUM contamination [20]. Fumonisins structurally resemble sphingolipids and can inhibit de novo sphingolipid biosynthesis by potently inhibiting ceramide synthases [24]. The most prevalent member of the FUM family is fumonisin B_1_ (FB_1_) [25].

Ochratoxins (OT) are produced by various *A.* spp. and *Penicillium* spp. The most abundant and harmful of the OT is OTA, which is primarily produced by *A. ochraceus* and *P. verrucosum* [21, 26]. Ochratoxin A inhibits protein synthesis, disrupts cell-cycle progression, and induce DNA adducts. It also inhibits ATP production and induce production of reactive oxygen and nitrogen species in mitochondria [27–29].

Due to the global occurrence of these mycotoxins and animal health risks concerning the intake of mycotoxins, the level of these mycotoxins in cereals and feed are currently regulated in a number of countries around the world [30]. Table 1 summarized the global occurrence of these mycotoxins as well as action levels and advisory/guideline limits set by European Commission, United States Food and Drug Administration and Canadian Food Inspection Agency for maize and maize products as well as finished feed for the animal species commonly addressed in the present review.

**Table 1:** Permitted/guidance levels of regulatory bodies for selected mycotoxins

Mycotoxin	Commodity	European Commission permitted/guidance level, mg/kg^a^	Food and Drug Administration permitted level/guidance level, mg/kg^b^	Canadian Food Inspection Agency permitted level/guidance level, mg/kg
Aflatoxin B_1_ (AFB_1_)	Maize	AFB_1_: 0.02	Aflatoxins (AF): 1. Immature animals: 0.02 2. Dairy animals: 0.02 3. Breeding swine and mature poultry: 0.1 4. Finishing swine 100 pounds or greater in weight: 0.2	AF: 0.02
	Maize silage			
	Finished feed	AFB_1_: 1. Dairy cattle and calves, dairy goats, piglets, and young poultry animals: 0.005 2. Cattle (except dairy cattle and calves), pigs (except piglets) and poultry (except young animals): 0.020	n/a	n/a
Deoxynivalenol	Maize	8	1. Swine (Grain and grain by-products not to exceed 20% of diet): 5 2. Chickens (Grain and grain by-products not to exceed 50% of diet): 10 3. Ruminating dairy cattle (> 4 months; 88% dry matter basis): 10 4. Other reviewed species (grain and grain by-products not to exceed 40% of diet): 5	n/a
	Maize Silage			
	Finished feed	1. Pigs: 0.9 2. Calves (< 4 months): 2 3. Other reviewed species: 5	Ruminating dairy cattle (> 4 months): 5	1. Cattle and poultry: 5 2. Swine, young calves and lactating dairy animals: 1
Fumonisins (FUM)	Maize	60	1. Swine (no more than 50% of diet dry weight basis): 20 2. Breeding poultry and breeding ruminants (including lactating dairy cattle) (no more than 50% of diet dry weight basis): 30 3. Poultry being raised for slaughter (no more than 50% of diet dry weight basis): 100	n/a
	Maize Silage			
	Finished feed	1. Pigs: 5 2. Poultry, calves (< 4 months): 20 3. Adult ruminants (> 4 months): 50	n/a	n/a
Zearalenone	Maize	2	n/a	n/a
	Maize Silage			
	Finished feed	1. Piglets and gilts (young sows): 0.1 2. Sows and fattening pigs: 0.25 3. calves, dairy cattle and goats: 0.5	n/a	1. Gilt diets < 1–3 2. Cow diets 10 (1.5 if other toxins present) 3. Pigs: < 0.25-0.5
Ochratoxin A	Maize	0.25	n/a	n/a
	Maize Silage			
	Finished feed	1. Pigs: 0.05 2. Poultry: 0.1	n/a	1. Swine diets (kidney damage): 0.2 2. Swine diets (reduced weight gain) 2 3. Poultry: 2

^a^FUM: Sum of fumonisin B_1_ and B_2_; ^b^FUM: Sum of fumonisin B_1_, B_2_ and B_3_

## Nutritional impact of major mycotoxins

Carbohydrates, lipids, and proteins are the major constituents of feed and serve as fuel molecules for animals as well as building blocks for growth and development of essential cellular components. The digestion of these nutrients and the subsequent absorption of the digestive end-products make it possible for cells and tissues to utilize them for proper functionality. Efficient utilization of these macronutrients is crucial to food animal production. Non-ruminant animals are generally considered more susceptible to nutritional effects of mycotoxins compared to ruminants [31, 32]. As a result, more research has been dedicated to poultry and swine than to ruminants.

### Feed intake

Feed intake is one factor affecting nutrient availability [33]. Effects of various mycotoxins on feed intake have been documented for various animal models (Table 2). However, limited data on FUM and OTA has been reported compared to AF, DON and ZEN. Effects of AF and DON on feed intake have been reportedly pronounced over a wide range of concentrations compared with ZEN and FUM (Table 2). However, concentrations of individual mycotoxins used in the reviewed studies that affected feed intake were all above the permitted/guidance levels of regulatory bodies where available (Table 1). The concentration of AF that reportedly reduced feed intake in poultry was as low as 0.04 mg/kg of feed [34] in broilers; whereas the lowest observed concentration of DON in pigs was 1.7 mg/kg of feed [53]. Moreover, co-occurrence of various mycotoxins has also been reported to reduce feed intake. With the concentrations of each individual mycotoxin respectively in accordance with legislated levels (Table 1), combinations of DON (5 mg/kg of feed) + FUM (20 mg/kg of feed) + ZEN (5 mg/kg of feed) was reported to result in reduced feed intake in broiler chickens, which was more pronounced than DON (5 mg/kg feed) alone [49]. Synergistic effects of combined DON (1 mg/kg) + ZEN (0.27 mg/kg) on feed intake were also reported in piglets compared with either DON or ZEN alone [52]. Furthermore, a large number of reviewed articles used either artificially contaminated experiential diets or purified powder rather than naturally contaminated diets (Table 2).

**Table 2:** Effects of selected major mycotoxins on feed intake

Mycotoxin	Source of contamination	Animal model	LOAEL, mg/kg feed^a^	NOEL, mg/kg feed^b^	Feed intake^c^	References
AFB_1_	Artificial	Broilers	0.04	NA	D	[34]
AFB_1_	Artificial	Broilers	NA	0.5	NS	[35]
AFB_1_	Artificial	Broilers	NA	1	NS	[36]
AFB_1_	Artificial	Broilers	5	NA	D	[37]
AF	Natural	Laying hens	0.123	NA	D	[38]
AF	Natural	Laying hens	0.267	NA	D	[39]
AFB_1_	Natural	Ducks	NA	Up to 0.1	NS	[40]
AFB_1_	Natural	Ducks	0.12002	NA	D	[41]
AFB_1_	Artificial	Ducks	0.21	NA	D	[42]
AF	Artificial	Turkeys	0.05	NA	D	[43]
AF	Artificial	Turkeys	0.5	NA	D	[44]
AF	Artificial	Pigs	NA	0.02	NS	[45]
AFB_1_	Artificial	Pigs	NA	0.28	NS	[46]
AFB_1_	Artificial	Dairy goats	NA	0.05	NS	[47]
DON	Artificial	Ducks	NA	5	NS	[48]
DON	Artificial	Broilers	NA	5	NS	[49]
DON	Natural	Broiler	NA	5	NS	[50]
DON	Artificial	Turkey	NA	5	NS	[51]
DON	Natural	Pigs	NA	1	NS	[52]
DON	Natural	Pigs	1.7	NA	D	[53]
DON	Natural	Pigs	2.86	NA	D	[54]
DON	Natural	Pigs	3.02	NA	D	[55]
DON	Artificial	Pigs	3.8	NA	D	[56]
DON	Natural	Pigs	4	NA	D	[57]
DON	Not specified	Pigs	5	NA	D	[58]
FUM	Natural	Broilers	NA	Up to 5.5	NS	[59]
FUM	Artificial	Broilers	NA	20	NS	[49]
FUM	Artificial	Ducks	NA	20	NS	[48]
FUM	Artificial	Turkey	NA	20	NS	[51]
FUM	Natural	Pigs	30	NA	D	[60]
FUM	Natural	Pigs	60	NA	D	[60]
OTA	Natural	Broilers	NA	0.172	NS	[61]
OTA	Artificial	Broilers	NA	2	NS	[62]
OTA	Not specified	Pigs	0.4	NA	D	[63]
ZEN	Artificial	Ducks	NA	0.5	NS	[48]
ZEN	Artificial	Turkey	NA	0.5	NS	[51]
ZEN	Artificial	Laying hens	NA	0.26	NS	[38]
ZEN	Artificial	Broilers	0.5	NA	D	[49]
ZEN	Natural	Pigs	NA	1	NS	[52]
ZEN	Purified powder	Pigs	NA	1	NS	[64]
ZEN	Purified powder	Pigs	NA	1.04	NS	[65]
ZEN	Purified powder	Pigs	NA	1.22	NS	[66]
AFB_1_ + OTA	Artificial	Broilers	0.025+ 0.1	NA	D	[67]
AFB_1_ + OTA	Purified	Dairy goats	NA	0.05 + 0.1	NS	[47]
AF + ZEN	Natural	Laying hens	0.123 + 0.260	NA	D	[38]
AFB_1_+ ZEN	Purified	Dairy goats	NA	0.05 + 0.5	NS	[47]
DON+FUM	Artificial	Dairy cattle	NA	0.733 + 0.994	NS	[68]
DON + ZEN	Natural	Pigs	0.2654 + 1	NA	D	[52]
DON + ZEN	Natural	Dairy cattle	NA	1.966 + 0.366	NS	[69]
DON + ZEN	Natural	Dairy cattle	NA	5.24 + 0.66	NS	[70]
ZEN + FUM	Purified ZEN+ Natural FUM	Broilers	NA	1+3.15	NS	[59]
ZEN + FUM	Purified ZEN+ Natural FUM	Broilers	NA	1+5.5	NS	[59]
DON + FUM + ZEN	Artificial	Ducks	NA	5 + 20 + 0.5	NS	[48]
DON + FUM + ZEN	Artificial	Turkey	NA	5 + 20 + 0.5	NS	[51]
DON + FUM + ZEN	Artificial	Broilers	5 + 20 + 0.5	NA	D	[49]
DON + FUM + ZEN	Artificial	Pigs	0.9 + 5 + 0.1	NA	D	[71]
AFB_1_ + OTA + ZEN	Purified	Dairy goats	0.05 + 0.1 + 0.5	NA	D	[47]

^a^*LOAEL* Lowest-observed-effect-level, *NA* not applicable, ^b^*NOEL* No-observed-effect level, *NA* Not applicable, ^c^*D* decreased feed intake, *NS* Not significantly affected

The reduced feed intake has been reportedly associated with feeding behavior changes in animals, such as reduced meal frequency and size, slower feeding rate [55, 72–74]. Different mechanisms of feed refusal may be involved. For example, the increased anorexia responses induced by TRC especially DON has been well documented mostly in mouse/mink model [75], but has been confirmed with pig models. DON can reduce feed intake by direct regulation of anorexigenic pathways in central nervous system after crossing blood-brain barrier in pigs [75, 76] or indirect peripheral regulations such as stimulating secretion of gut-satiety hormones such as peptide YY and cholecystokinin in pigs [56, 77, 78]. Other mechanisms such as mycotoxin-induced gut dysbiosis [79] and secretion of inflammatory mediators [80] could also contribute to behavior changes and feed refusal in animals.

Although mycotoxin-induced anorexia has been evidenced, especially for DON, several studies have reported progressive disappearance of negative effects on feed intake over time in pigs either continuously on DON-contaminated diets [54] or on a normal DON-free diets after initial exposure to DON in the experiments [81], with the depression in feed intake being most severe usually within 2 weeks after exposure [54, 55, 58, 77, 81]. The former scenario suggested that animals appeared to show tolerance to the presence of DON in the diet [54, 55, 58]. Although such adaptive mechanisms of pigs have not been fully understood, this could be linked to the possible alteration in intestinal microbes to favor detoxification of DON in pigs. However, piglets fed with diets contaminated with combined low-concentration DON (1 mg/kg feed) and ZEN (0.27 mg/kg feed) were reported to fail to recover after the mixture was withdrawn from the diets [52]. High concentration of FUM (58 mg/kg feed) also resulted in the failure of recovery in pigs compared to control group on mycotoxin-free diet [60].

The feed intake of ruminants has not been observed to be affected by DON and ZEN [68–70], whereas it was reportedly reduced by the mixture of AFB_1_, OTA and ZEN in lactating goats [47].

### Nutrient digestibility

Reduced ability of food animals to efficiently utilize their feed has been documented (Table 3). Several reviewed articles reported decreased digestibility of dry matter, gross energy and/or metabolizable energy, crude protein/amino acids, crude fat of animals after exposure to either AF, DON or FUM alone as well as combination of multiple *Fusarium* mycotoxins in poultry and pigs (Table 3). The reduced digestibility of neutral detergent fiber was also reported after lactating cows were exposed to *Fusarium* mycotoxins below the EU maximum levels [68]. Previous studies indicated that lower levels of FUM (5.5 mg/kg feed) [59] in broiler chickens and ZEN (1 mg/kg feed) in pigs [64], respectively, had no effects on digestibility of dry matter, crude protein and gross energy. Most of concentrations that negatively affected nutrient digestibility were above legislated maximum levels (Tables 1 and 3). However, several studies respectively reported that reduced nutrient digestibility in poultry, pigs and dairy cows was observed after animals were exposed to DON or FUM alone [85] or combinations of *Fusarium* mycotoxins [59, 68, 85] at concentrations in compliance with the regulations (Table 1). There is variability of results reported regarding the effects of different mycotoxins on nutrient digestibility, which could be due to the factors outlined in Table 4.

**Table 3:** Effects of selected major mycotoxins on nutrient digestibility

Mycotoxin	Source of contamination	Animal model	LOAEL, mg/kg feed	Digestibility	Reference
AFB_1_	Natural	Ducks	0.12002	DM, CP, GE	[41]
AFB_1_	Artificial	Broilers	0.04	DM, CP, GE	[34]
AF	Artificial	Broilers	0.5	Net protein utilization;	[82]
AFB_1_	Artificial	Broilers	1.5	ADE, N and amino acids	[83]
AF	Artificial	Broilers	2	Protein efficiency, net protein utilization	[82]
AF	Artificial	Laying hens	0.6	ADE and AME	[84]
AFB_1_	Artificial	Pigs	0.28	DM, GE, ether extract	[46]
DON	Natural	Pigs	2.86	DM, GE, CP	[54]
DON	Natural	Pigs	4	DM, fat, Energy	[57]
DON	Artificial	Broilers	5	Tyrosine	[85]
DON	Artificial	Pigs	11.2	Tryptophan	[86]
FUM	Artificial	Broilers	20	DM	[85]
FUM	Artificial	Pigs	15	CF, DE	[87]
FUM	Artificial	Pigs	30	CF	[87]
OTA	Artificial	Broilers	2	Protein efficiency, netprotein utilisation	[82]
ZEN	Artificial	Pigs	11.6	Tryptophan	[86]
ZEN + FUM	Purified ZEN+ Natural FUM	Broilers	1 + 3.15	DM, OM, CP, GE	[59]
ZEN + FUM	Purified ZEN+ Natural FUM	Broilers	1 + 5.5	DM, OM, CP, GE	[59]
DON + FUM	Artificial	Broilers	1.5 + 20	DM	[85]
DON + FUM	Artificial	Broilers	5 + 20	DM, ADE	[85]
DON + FUM	Artificial	Dairy cattle	0.733 + 0.994	DM and neutral detergent fiber	[68]

^a^*LOAEL* Lowest-observed-effect-level, ^b^*DM* Dry matter, *CP* Crude protein, *GE* Gross energy, *DE* Digestible energy, *OM* Organic matter, *ADE* Apparent digestible energy

**Table 4:** Factors contributing to viability of results in the literature

	Factor	Example	References
Diet-related factors	Mycotoxin	Aflatoxins, deoxynivalenol, fumonisins	[36, 48, 56]
	Source of contamination	Naturally contaminated	[52]
		Artificially contaminated	[88]
		Purified powder	[66]
	Substrate of contamination	Corn	[54]
		Wheat	[58]
		Barely	[52]
	Level of mycotoxin concentration	A wide range of mycotoxin concentrations	[54, 56, 58]
	Individual or combined contamination	Synergistic effects of deoxynivalenol and fumonisins	[89]
Animal-related factors	Species	Poultry, pigs, dairy cattle, dairy goats	[35, 46, 47, 68]
	Breed	Pigs: Yorkshire × Chester White × Duroc, Landrace × Yorkshire, Camborough Plus × C337	[52, 58, 90]
		Broilers: Arbor Acres, Ross-308	[34, 35]
		Ducks: Cherry Valley ducks, Pekin	[41, 42]
		Laying hens: Hy-Line Brown laying hens, ISA Brown laying hens	[38, 39]
	Sex	Pigs: all males, mixed sex and all females	[54, 56, 57]
	Age	21-d-old and 42-d-old female pigs	[52, 65]
	Production stage	Early-lactation vs. mid-lactation cows	[68, 69]
	Diet composition	Forage types in dairy total mixed ration	[68, 69]
Other factors	Exposure duration	Pigs: 21-d, 35-d test period	[52, 65]

### Digestive and absorptive processes

Nutrient digestion and absorption are vital for energy maintenance and nutrient homeostasis and require a network of biochemical reactions and biomolecules in various tissues to ensure requirements for productive purposes are met. The intestine is the renowned site for nutrient digestion and absorption. Distinct finger-like projections in the small intestine called villi increase the absorptive surface area by extending into the lumen. The structure and functionality of the intestine epithelium is maintained by continuous renewal and differentiation of intestinal epithelial cells arising from crypt stem cells [91]. As chyme or digesta passage through the small intestine, brush border (BB) enzymes including oligopeptidases, lipase and oligosaccharidases, are responsible for the final stage of luminal nutrient digestion prior to nutrient absorption. These enzymes can also further hydrolyze the fraction of undigested nutrient oligomers following buccal, gastric, and pancreatic digestion [92]. Following digestion, uptake and absorption of glucose,amino acids and fats is facilitated by several transporter proteins located within brush border membranes [93, 94]. Although, the primary site of nutrient absorption takes place in the small intestine [95], the contribution of the pancreas should not be overlooked. The pancreas can synthesize digestive enzymes such as α-amylase, lipase and proteolytic enzymes that empty into the duodenum; these enzymes respectively digest starches, fats, and proteins [96].

#### Morphology of the pancreas and the intestine

The integrity of tissues is crucial to their functionality. Despite the importance of pancreas, the effects of mycotoxins on pancreatic function are limited. AFB_1_ reportedly alters morphology of pancreas in poultry [97, 98], and increased the relative weight of pancreas in ducks and broiler chickens [99, 100]. However, Matur et al. [101] did not observe any effects of AFB_1_ on relative pancreas weight in breeder hens, but the authors suggested that was due to the low concentration of AFB_1_ that was used in their study.

Several studies have demonstrated that mycotoxins altered the intestinal morphology. The intestinal villus height and crypt depth, and sometimes the ratio of villus height to crypt depth (H/D), are used as morphological indicators of the likely digestive and absorptive capacity of the small intestine; an increase in H/D ratio corresponds to an increase in digestion and absorption [102]. AFB_1_ at concentrations of 0.15 mg/kg, 1.2 mg/kg and 2 mg/kg feed reportedly reduced villus height, increased the crypt depth in ducks [41], laying hens [84] and broiler chickens [103], respectively. Ochratoxin A at low level of 0.05 mg of OTA/kg body weight/day also decreased H/D ratio in broiler chickens [104]. The alteration in morphology of intestine has also been observed in broiler chickens [49] and turkeys [105] after birds exposed to 5 mg DON/kg feed and 4.5 mg DON/kg feed, respectively; both concentrations were in accordance with legislated levels (Table 1). In terms of pigs, it has been previously reported that DON above 3 mg/kg feed could result in shorter villi [54, 57, 106]. However, this concentration range of DON was all above permitted levels regulated by the legislative bodies (Table 1). Data on effects of FUM and ZEN on the intestine is limited. Previous study indicated that co-occurrence of FUM (6 mg/kg feed) + DON (3 mg/kg feed) rather than FUM (6 mg/kg feed) alone affected villus height or crypt depth, suggesting synergistic effects of these two mycotoxins [89]. Metayer et al. [49] reported that FUM (20 mg/kg) and ZEN (0.5 mg/kg) within EU regulated levels (Table 1) increased the crypt depth in broiler chickens and the mixture of DON (5 mg/kg) + FUM (20 mg/kg) and ZEN (0.5 mg/kg) also altered intestinal morphology in broiler chickens.

The intestinal epithelium is the first site of exposure following mycotoxin ingestion and may be exposed to higher concentrations than other tissues [17]. Changes in villus height reflect the balance between intestinal epithelial cells (IEC) proliferation and apoptosis [89, 107], which is in line with findings that several mycotoxins can cause oxidative stress induced IEC apoptosis and cell cycle arrest both in vivo and in vitro [108–110]. Previous studies suggested effects of mycotoxins on the intestinal morphology may appear to be section-specific, with effects on duodenum and jejunum being more pronounced than ileum [49, 57, 89, 111]. This could be due to the majority of the ingested mycotoxins being absorbed in the upper part of the intestine [17].

#### Digestive enzymes

Different studies have reported an increase in activity of pancreatic α-amylase, lipase, trypsin, and chymotrypsin across several poultry species such as broilers, breeder hens and ducks after exposure to different AFB_1_ levels [42, 101, 112]. These authors suggested that the increased activities of α-amylase and lipase were abnormal and pathologic, which may be ascribed to increased pro-enzyme released from the injured pancreas [42, 101, 112]. The results from previous study also suggested that effects of AF on pancreatic enzymes could be concentration- and time-dependent [112]. For example, the activity of α-amylase was elevated as the level of AF increased after 2 weeks animals were on the test diets [112]. Moreover, AF at 2.8 mg/kg feed started to induce increased activity of α-amylase and lipase on d 14; whereas trypsin activity was not affected until d 35 in the experiment [112].

Only limited studies have analyzed the activity of pancreatic digestive enzymes that has been secreted into small intestine, however, contradictory effects were reported. For example, Han et al. [99] reported increased enzyme activities of lipase and α-amylase in the duodenum in ducks exposed to 0.04 mg AFB_1_/kg feed. Increased level of proenzymes released from injured pancreas could possibly account for this finding [99]; the increased activities may also be due to a compensatory effect of the birds to meet their nutrient needs in response to reduced feed intake [42]. In contrast, Matur et al. [101] observed decreased activities of decreased α-amylase and lipase in breeder hens fed diet containing 0.1 mg AF/kg feed [101]. The authors suggested there could be a secretion problem into duodenum from damaged pancreas [101].

With regards to BB digestive enzymes, Applegate et al. [84] reported that activity of intestinal maltase exhibited hermetic response pattern. Specifically, the activity was increased in laying hens by feeding up to 1.2 mg/kg of purified AF and declined at 2.5 mg/kg [84]. These findings could be explained by the phenomenon called “hormesis”, which is an adaptive beneficial effects that occurs in cells or living organisms after exposure a low concentration of a chemical agent or environmental factor that is damaging at higher concentrations [113]. It is considered an adaptive compensatory process following an initial disruption in homeostasis [113], and its detection is highly dependent on experimental design [114]. Biphasic effects of AFB_1_ on body weight [115] and immune response [116] in chickens has been reported. Several other studies consistently reported no effects of AFB_1_ on sucrase and maltase activities in poultry [41, 42, 83]. Moreover, both DON (1 mg/kg feed) and ZEN (1.04 mg/kg feed) were respectively reported to decrease the activities of sucrase, maltase and lactase in pig intestine [65, 117]. The results from previous study could indicate that DON selectively affect enzyme activities in different sections of the intestine, implicating effects of DON on BB enzymes could be segment-dependent in the intestine [117].

#### Nutrient uptake

Dietary nutrients can only be utilized by different animal tissues after they have been transported across the intestinal epithelium and entered systemic circulation. Different studies have been carried out to investigate the effects of mycotoxins on glucose and amino acid transport across the small intestine using short-circuit current (Isc) measurement, which is a measure of ion transmembrane flux [118], and is a good indicator of sodium-dependent glucose and/or amino acid transport [17]. Awad et al. [119] reported that the Isc induced by the addition of glucose was reduced in broiler chickens fed with a DON-contaminated diet containing 10 mg/kg, suggesting disrupted glucose uptake induced by DON. Similar inhibitory effects of DON on glucose-induced Isc were also observed in laying hens in other studies [120, 121]. This inhibitory effects of DON on Isc could be attributed to its strong inhibition on sodium-dependent glucose co-transporter (*SGLT1*) [121], which could be supported by the reduced mRNA expression of *SGLT1* and the facilitated glucose transporter *GLUT2* observed in broiler chickens fed diets naturally contaminated with 1 mg/kg and 5 mg DON/kg feed later in another study [50]. Recently, downregulation of mRNA expression of facilitated glucose transporter *GLUT1* and several amino acid transporter including peptide transporter *(PepT1)* and Heavy chain corresponding to the b^0,+^ transport system *(rBAT)* was reported in broiler chickens after exposure to 4-10 mg DON/kg feed [111, 122]. In pigs exposed to 2.86 mg DON/kg, mRNA expression of *PepT1* and *SLUT1* was not reportedly affected [54].

Aflatoxins also appeared to affect glucose and amino acid transport across the small intestine. Exposure to 1.5 mg/kg AFB_1_ had no effect on mRNA expression of *SGLT1* and *GLUT2* in broiler fed diets artificially contaminated with AFB_1_; however, an increase in mRNA expression of several amino acid transporters including *b*^*0,+*^*AT*, *EAAT3*, *PepT1*, *rBAT*, *yLAT1*, and *yLAT2* was observed after exposure to AFB_1_, which might be a compensatory response for amino acid deficiency and impaired protein activities; this may also suggest an increased requirement for amino acid absorption for the subsequent protein synthesis [83].

These results suggested that inhibitory effects of DON and AFB_1_ on mRNA expression of glucose and animal acid transporters could contribute to the adverse nutritional impact on food animals. However, more relevant studies need to be carried out to better understand mycotoxin-induced nutrient uptake disruption.

#### Nutrient metabolism by gut microbiota

The intestine, especially large intestine, is a complex ecosystem comprised of trillions of microbes. These microbes play multifaceted role in maintaining health, including providing nutrients, metabolizing complex food sources and toxins, and facilitating normal development of neonatal intestinal immune function and its maintenance throughout life [123–125]. Microbiota residing in the large intestine are mainly responsible for the digestion of dietary substrates that escaped proximal digestion in the gastrointestinal tract; such digestion also provides nutrients and energy sources like short chain fatty acids, essential amino acids and vitamins to the host [126, 127]. Recently, a study conducted by Wu et al. [111] using 16S rRNA gene amplicon sequencing demonstrated that exposing broiler chickens to DON at 10 mg/kg significantly decreased the abundance of caecal microbiota, namely the Proteobacteria (phylum level), *Escherichia* and *Cc-115* (genus level), as well as the *Escherichia coli (*species level*)* that are beneficial to nutrient utilization processes such as digestion and absorption of protein, lipid and carbohydrates. Contamination of DON also reportedly tended to reduce other microbes including *Lactobacillus* and *Prevotella* (genus level), *Ruminococcus bromii, Desulfovibrio*, *C21_c20*, and *Eubacterium dolichum* (species level).

Nutritionally similar to intestinal microbes, the ruminal microbiota also has the capacity to convert recalcitrant fibrous plant material into assimilable energy and nutrients for ruminant species, and also contributes to rumen epithelium development and establishment of the immune system [128]. Different studies have been conducted in vitro with rumen fluid to evaluate the nutrient metabolism in rumen. Boguhn et al. [129] reported DON at 5 mg/kg in the diet failed to alter the ability of rumen microbes to ferment organic matter and carry out protein synthesis. In contrast, another study using rumen fluid containing DON at 40 mg/kg reported a reduction in gas production, ammonia-N and volatile fatty acids (VFA) concentrations [130]. AFB_1_ in rumen fluid at various concentrations has also been reported to reduce digestibility of DM, gas production and concentrations of ammonia-N and VFA [131–133].

**Fig. 1:**
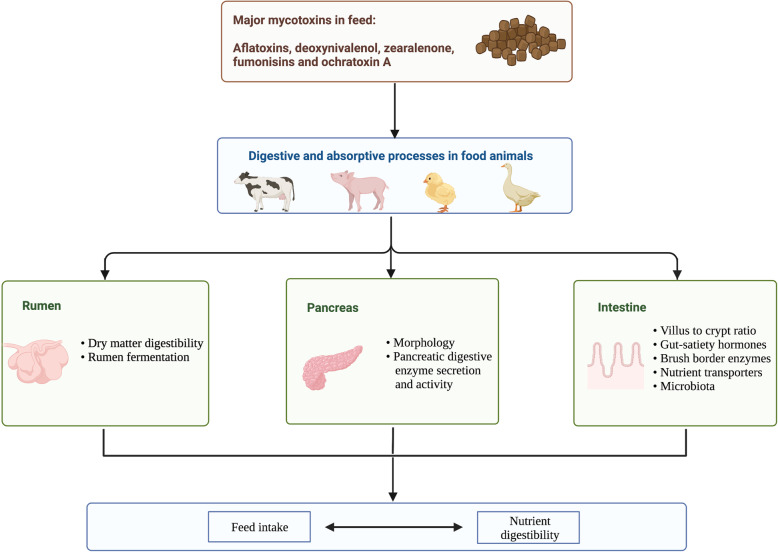
Schematic diagram depicting physiological impact of mycotoxins on different organs affecting nutrient utilization

Taken together, data suggest that mycotoxins can negatively impact feed intake, nutrient digestion, and absorption, thus making nutrients less available for animals to utilize for productive purposes (Fig 1).

## Differences in susceptibility to mycotoxin exposure

Mycotoxin-related impacts reported in the literature vary greatly. This could be ascribed to several factors that has been summarized in Table 4. Among which, species, age and sex of animals could be major contributing factors accounting for differential susceptibility of animals to mycotoxin exposure from the following aspects.

### Microbial transformation of mycotoxins in the gastrointestinal tract

Microbial metabolism of mycotoxins could affect bioaccessibility of parent mycotoxins in animals of different species. Maresca et al. [134] proposed that localization of resident microbiota in the gastrointestinal tract play a role in resistance of animals to mycotoxins. This author categorized animals into two groups: 1) animals having large numbers of microbes located before and after the small intestine (polygastric animals such as ruminants that have microbes in the rumen and in their colon, and birds that have microbes in their crop and cecum), and 2) monogastric species including humans, pigs and rodents that have a large number of microbes located only after the small intestine. On this basis, ruminants are more resistant to mycotoxins compared to monogastric animals in part because most mycotoxins are transformed to less or non-toxic derivatives by microbes in the rumen [31, 32]. This could be exemplified by the transformation of DON and OTA by rumen microbes into their less toxic derivatives deepoxy-deoxynivalenol (DOM-1) and OTA-α, respectively [135]. Resistance of poultry to DON is another case in point. Poultry species are thought to be less susceptible to DON than swine [118, 134] because microbes in the crop transform DON to its less toxic derivative DOM-1 and deepoxy-DON-3-sulfate (DOM-3-sulfate) [134, 136]; this reduces the amount of DON reaching the lower gut. Absence of pre-intestinal microbial transformation in pigs means a larger amount of DON reaches the lower porcine gut.

In addition to the localization of microbiota, the microbial diversity could also be a contributing factor to age-dependent mycotoxicity. The effects of DON and FB_1_ have been reported to be more pronounced in young pigs [137], which lack microbial diversity. Indeed, the microbial complexity is thought to increase as animals age [138, 139].

### Mycotoxin metabolism in animals

The mechanisms by which mycotoxins are metabolized by animals following absorption across the intestinal epithelium could also contribute to species-specific sensitivity to mycotoxins. Detoxification processes that biotransform mycotoxins involve phase I and phase II metabolizing enzymes present both in the intestine and the liver [134, 140]. These biotransformation pathways vary across species, in part, due to species-specific types and activities of metabolizing enzymes [118, 141, 142]. The domestic turkey for example, is one of the most susceptible farm animals to AFB_1_, and this susceptibility is mainly associated with a combination of efficient hepatic bioactivation of AFB_1_ to its highly toxic metabolite exo-AFB_1_-8,9-epoxide (AFBO) by cytochromes P450 (CYP) enzymes, members of the phase I metabolizing enzymes and subsequent deficient inactivation and detoxification of AFBO through conjugation mediated by the phase II enzymes hepatic glutathione–S transferases (GST). In contrast, pigs are more resistant to AFB_1_ toxicity in comparison to turkeys due to their efficient conjugation of AFBO by GST and subsequent excretion of these AFBO conjugates [140, 141]. Interestingly, in the context of ZEN, pigs are more susceptible to ZEN compared with poultry due to greater production of the more toxic α-ZOL metabolite produced by CYP and rather low expression of UDP-glucuronosyltransferases (UGT) phase II metabolizing enzymes that are responsible for subsequent conjugation of α-ZOL and its inactivation [143].

The differences in mycotoxin metabolizing mechanisms could also explain age-dependent difference in mycotoxin toxicity. Since the effect of mycotoxins on growth was reported to be greater in younger animals of pigs and poultry compared to older animals [144, 145], it is possible that differences in detoxification activity and quantity of hepatic enzymes could contribute to the age-dependent differences in sensitivity to mycotoxins. In support of this, it has been reported that activity of CYP enzymes that are responsible for metabolizing AFB_1_ is inversely related to age with regards to young poults and chickens [140]. Also, in rats it has been reported that lower levels of AF–glutathione transferase conjugate can be detected in young animals compared to their adult counterparts, suggesting lower capacity for detoxification [145]. Collectively, these studies suggest the liver of young animals is less efficient at biotransforming mycotoxins for elimination, which could contribute to higher susceptibility of young animals to mycotoxicosis. The activities of metabolizing enzymes could also be sex dependent. Studies showed that there were sex-dependent differences in the activities of certain CYP enzymes in human liver and mice [146–149], which suggests animals of different sexes used in the experiments could lead to varying response to mycotoxin exposure.

### Other factors

Other factors may also contribute to age- and species-specific susceptibility to mycotoxins. Variation in the number and affinity of oestrogen receptors for ZEN may affect susceptibility to this mycotoxin. For example, pigs and sheep are the highly susceptible species, and immature animals are generally considered to be more susceptible than adults [14]. Different transport mechanisms and cellular uptake within renal tissue has also been demonstrated to affect OTA nephrotoxicity [14]. Also, gastrointestinal transit time can influence exposure duration of the gut to unabsorbed or poorly absorbed mycotoxins such as FB_1_ [17, 32]. Induction of oxidative stress by mycotoxins have been shown to be implicated in their toxicities [150, 151]. Upon ingestion of mycotoxin, the ability of animals to alleviate oxidative stress may affect their susceptibility to mycotoxin toxicity. Antioxidant capacity has been reported to vary depending on age and sex [152–154], which could also be a contributing factor to possible variations resulting from different ages and sexes used in the studies.

## Mycotoxin risk management

### Prevention strategies

Mycotoxin contamination can occur at any stage of feed supply chain including crop cultivation, harvest, storage and distribution of crops and compound feeds. Preventative measures should be taken to minimize mycotoxin contamination. Predicting the risk of mycotoxin contamination pre-harvest in cereal crops is a useful and effective tool for pre-harvest and post-harvest mycotoxin management [155]. These predictive models usually use an empirical or mechanistic approach to quantify mycotoxins, and some of them have been implemented in agricultural sectors across Europe to support food source decision making for farmers [156]. Recently, a machine learning approach has been incorporated to build mycotoxin prediction models [156, 157], which could be a promising contribution to mycotoxin control.

Good agricultural practices are one of the primary pre-harvest strategies for mycotoxin prevention are mainly performed at the level of crop cultivation. These practices could include implementing breeding programs for selecting more mycotoxin-resistant plants, crop rotation, soil, and irrigation management, use of registered fungicides and insecticides for control of mild and insect infestations [21]. Application of non-aflatoxigenic *Aspergillus flavus* has been accelerated as biological control to mitigate pre-harvest aflatoxin contamination over the past years [158]. Post-harvest storage management is also crucial to counteract mycotoxin contamination. Techniques, such as maintaining low moisture levels (less than 15%) and low temperature in the storage environment as well as preserving the integrity of whole grains, are crucial to controlling the level of fungi and mycotoxin contaminants [159].

### Decontamination strategies

When prevention is not achievable, it is important to apply strategies to mitigate mycotoxin contamination of feed ingredients or compound feeds. Mitigating mycotoxins should be carried out at an integrated level throughout the supply chain to ensure mycotoxin concentrations in feed are compliant with legislated maximum tolerated/recommended tolerance levels, and therefore feed products are considered safe for animal consumption. Detection of mycotoxins in the cereals and animal feeds using different technologies is critical for monitoring mycotoxin occurrence and mitigation [160]. In terms of efficiency and feasibility, mitigation approaches should include mycotoxin removal and inactivation strategies that do not lead to the production of toxic residues or jeopardize the nutritive value and other desirable parameters, such as palatability, of products [161]. It should also be noted that mitigation needs to be simple and inexpensive to perform. Different remediation strategies that have been deployed in an attempt to mitigate the risk of mycotoxin contamination in feed and their effectiveness, as well as limitations, have been assessed below.

#### Physical techniques

##### Dilution

Dilution involves mixing mycotoxin-contaminated and uncontaminated grains to achieve a total mixture containing mycotoxin concentrations below the legislated maximum tolerated levels/recommended tolerance levels [4, 162]. Dilution is a simple and widely used economical approach for mitigating the risk of mycotoxin contamination in feed. However, success of this approach will depend on the degree of the contamination and the availability of uncontaminated grain sources. In some countries, such as European Union, this practice is no longer permitted [4, 162].

##### Grain cleaning and sorting

Certain steps of grain processing contribute to the decontamination of mycotoxins. Unprocessed cereal grains are normally received in bulk and often contain undesired materials such as dust, foreign materials and interior kernels [163, 164]. Broken and damaged kernels in these bulk loads usually contain most of the mycotoxin contaminants [164]. In large-scale feed manufacturing, the grain cleaning and sorting is applied to mechanically remove dust, foreign materials and interior kernels from healthy grains, mainly based on the lower density of potentially infected grains and feed contaminants [162, 163]. This cleaning and sorting practice have also been shown to reduce contamination of DON and some other mycotoxins such as nivalenol, T-2 and HT-2 in wheat and wheat cultivars [165, 166], as well as the contamination of AF [164, 167]. However, the reduction of mycotoxins by cleaning processes could be highly variable [168].

Milling is another grain processing step that could potentially mitigate mycotoxin contamination. The by-products derived from milling are used as raw ingredients in animal feed. Milling processes have been reported to redirect existing mycotoxins into different milled fractions rather than reduce mycotoxin contamination [162, 169, 170]. Fractions derived from outer layer of kernels tend to have higher mycotoxin concentrations than inner parts through milling processes, since outer parts of grains are more easily contaminated with mycotoxins [165, 166], therefore, depending on the milled fractions, milling could also result in reduced mycotoxin concentrations.

##### Thermal methods

Although most mycotoxins are generally thermally stable compounds, processes such as crumbling, pelleting and extrusion during feed manufacturing, combining high-speed shearing and superheated steaming, can reduce mycotoxin concentration, but these processes do not completely eliminate mycotoxins [163, 171]. These processes have been shown to reduce concentrations of AF, FUM, DON and ZEN [159, 162]. The degree of reduction depends mainly on several factors, including types of mycotoxins, initial mycotoxin concentration, exposure temperature and duration at high temperature, degree of heat penetration, moisture content and pH, among others [171–173]. In general, temperatures higher than 150 °C, long exposure time, high moisture content and low initial mycotoxin concentration all result in greater reduction in mycotoxin concentration [159, 171]. However, with many influencing factors involved, the effects of thermal processing on mycotoxin reduction can be quite variable [162, 163]. It may contribute to mycotoxin mitigation, but it alone is not sufficient for mitigation of exposure risk.

#### Chemical techniques

##### Chemical agents

A wide variety of chemical agents have been found to be effective to reduce the concentration of several mycotoxins in different commodities, including acids, bases, chlorinated substances, sodium metabisulfite, ammonia and dry ozone. However, toxic metabolites can be generated by chemical treatment and the nutritional value, and the palatability of the feed can be diminished. Handling of these chemicals also poses a potential risk to workers [30, 174]. The application of chemical agents for decontamination is currently not authorized within the EU and US [30, 162].

##### Irradiation

For many stored cereals, irradiation is used as an approach to reduce or eliminate fungi and other potential pathogens infecting the grains, and it can partially eliminate mycotoxins [21, 164]. Three sources of ionizing radiation are authorized in food or feed manufacturing in Europe, including gamma-radiation, X-rays, and electron beams [162]. It was reported that gamma-irradiation reduced AFB_1_ content in maize and chicken feed, respectively, and an increase in irradiation dose showed better reducing effect [175, 176]. There are, however, some concerns about use of irradiation, including public concern about the safety of ionizing irradiation, changes in nutritional value and added food processing cost [162, 177].

#### Mycotoxin-detoxifying agents as feed additives

Depending on the mode of actions, mycotoxin-detoxifying agents (MDA) can be categorized into biotransforming agents (BA) and adsorbing agents (AA) according to a scientific reported submitted to ESFA [178]. The BA, such as bacteria/fungi or enzymes, degrade mycotoxins into less toxic metabolites, whereas the AA migrate mycotoxin contamination by decreasing mycotoxin bioavailability, which leads to a reduction in mycotoxin uptake and distribution to the blood and target organs.

##### Mycotoxin biotransforming agents

Mycotoxin biotransforming agents include microorganisms or their enzymes, which biologically transform mycotoxins into non- or less-toxic metabolites via routes such as hydrolysis, de-epoxidation, acetylation, oxidation, ring/side chain cleavage, and glycosylation by acting molecular signatures on each mycotoxin that confer their toxic effects [30, 179]. Mycotoxin biotransforming microorganisms have been isolated from the environment (i.e., soil, cereal grains and insects) and resident gastrointestinal microbes from animals [174]. Their detoxifying capabilities have been reported to be effective for different mycotoxins such as AFB_1_, ZEN, OTA and FB_1_ [180, 181]. The *Eubacterium* BBSH 797 bacterial strain, originally isolated from bovine rumen fluid was the first microbe used as an MDA. This MDA is able to detoxify TRC and is now used commercially for mitigation [30, 182]. Results of animal trials have shown that *Eubacterium* BBSH 797 significantly reduce the adverse effects of DON on sows and dairy cows, and T-2 toxin on growing broiler chickens [179]. *Trichosporon mycotoxinivorans* is a yeast strain that has also been thoroughly investigated and it is also commercially used as an MDA. This yeast, isolated from the hind-gut of the termite *Mastotermes darwiniensis* can detoxify both ZEN and OTA [179, 183]. Application of enzymes that can biologically degrade mycotoxins is an attractive alternative to microorganisms as they catalyze chemical reactions in a highly specific and efficient manner and offer advantages in terms of safety and ease of handing compared to viable microorganisms but are restricted to a specific mycotoxin substrate [180]. Several enzymes being identified and reported to have the capacity to biologically degrade FUM, AFB_1_, OTA, ZEN and DON [180, 181]. An ESFA-approved enzyme-based feed additive has been used commercially, and shows efficient degradation of fumonisins in pigs and avian species [184, 185]

Certain criteria that have to be fulfilled for the effective use of microorganisms and enzymes make their industrial application quite complicated and limited. For example: 1) isolated microbial strains should be non-pathogenic; 2) the detoxification process should yield non- or less-toxic compounds compared to the parent mycotoxin, and the efficacy of these MDA including BA needs be assessed and proved using specific biomarkers for certain mycotoxin [184]; 3) the degradation processes should occur rapidly, and the BA should be able to survive, adapt and be stable under different oxygen conditions and pH levels in the complex environment of the gastrointestinal tract; 4) the organoleptic and nutritive properties of the feed should be preserved; 5) lastly, viability of isolated microorganisms, and detoxification activity of microbes and enzymes, should be maintained through feed processing methods and be stable in the final commercial products [180, 186]. Several other time-consuming and complex processes may also limit the industrial application of enzymes. These include degrading enzyme identification and characterization, detoxifying-enzyme isolation as well as the involvement of molecular engineering and structure-function modifications of native enzymes by targeted or random mutagenesis, since native enzymes usually do not respond to each distinctive requirement of a perfect industrial enzyme [180, 186].

##### Mycotoxin adsorbing agents

The other class of MDA is mycotoxin adsorbing agents or adsorbents (AA), which help to alleviate the harmful effects of mycotoxins in livestock and poultry through direct binding to mycotoxins; this decreases their bioavailability or reduces their intestinal absorption, promotes the formation of mycotoxin-adsorbent complexes, and their consequent excretion via the fecal route [178].

Mycotoxin adsorbents are divided into inorganic and organic AA. Inorganic AA, also known as mineral adsorbents, are mainly phyllosilicates of the clay mineral group and also include tectosilicates like zeolites and activated charcoal. Inorganic AA are considered first-generation AA [180]. The most significant AA among phyllosilicates group are bentonite, montmorillonite, hydrated sodium aluminosilicates and smectite. The binding capability of inorganic AA depends on the physio-chemical structures of both AA and mycotoxins; this includes the charge distribution of AA and mycotoxins, surface area and pore size of the AA, polarity, and shape of the mycotoxins [159, 187]. Some mineral AA such as bentonites, zeolites and activated charcoal have been reported to adsorb ZEN, OTA, FUM and DON in vitro, however, in vivo confirmation studies are lacking [30, 54]. Most inorganic AA have been recognized as efficient binders of AF, as supported by in vivo studies, but they appear to have very limited capability of binding to other mycotoxins such as ZEN, TRC and FUM [30, 180, 188, 189]. Unfortunately, mineral AA are also known to adsorb micronutrients and have negative effects on the bioavailability of vitamins, amino acids, and minerals in feed [188, 189]. Mineral AA also have ecological disadvantages since the degradation of bound mycotoxins after they have been excreted is relatively slow [162].

In an attempt to overcome this inefficacy of inorganic AA, organic adsorbents originating from cell wall components of microorganisms have been developed as second-generation AA [180]. Cell wall components from *Saccharomyces cerevisiae* yeast strain are commonly used organic AA. The major functional fractions of yeast cell wall (YCW) responsible for mycotoxin binding include β-D-glucan and mannan oligosaccharides, which bind to mycotoxins via hydrogen bonding and van-der-Waal forces [188, 190, 191]. The proportion of the functional organic AA varies with the microbial strains and processing [192, 193], and differences in product purity and supplemental concentration can lead to different efficacy since their affinity to mycotoxins is reversible and saturable [192]. Compared to its inorganic counterparts, the YCW has exhibited greater capacity of binding to a wider spectrum of mycotoxins such as DON, ZEN, OTA and AFB_1_, and alleviating negative effects of mycotoxins has been investigated in numerous scientific publication both in vitro [28, 190, 191, 194, 195] and in vivo using poultry, pigs and ruminants [28, 45, 192, 196–200]. Another advantage of the YCW products is that they are biodegradable, and therefore the toxin-binder complexes do not accumulate in the environment after being excreted in the feces [188].

## Conclusions and future directions

Feed is the only source of energy and nutrients for farm animals. Sound practice in nutrition is required for animals to achieve their genetically selected production potential. Various mycotoxins and their mixtures can lead to negative nutritional outcomes, which could limit nutrient and energy availability to farm animals, thus, resulting in a sub-optimal production performance. However, it is worth noting that nutritional impact should not be the only criterion for assessment of overall mycotoxicity to food animals as it is well documented mycotoxins could lead to other adverse effects in various organs and systems such as gastrointestinal tract, liver or kidney, as well as the nervous, reproductive and immune systems in food animals, in some cases without affecting growth performance [201].

Moreover, in modern highly intensive and large scaled livestock production, mycotoxin contamination in feeds is only one of the various challenges that food animals face, such as environmental, nutritional non-infectious or infectious stressors [202]. As a result, mycotoxins could pose a cconfounding effect on animal production. For example, it has been reported that exposure to most *Fusarium* mycotoxins increases animal susceptibility to infectious diseases such as coccidiosis in poultry and swine respiratory diseases [203].

Although various mycotoxin management measures are available, there is still room for improvement given the challenges encountered in mycotoxin management. More effort should be made on assessment of emerging and modified mycotoxin occurrence and their toxicity (toxicokinetic and toxicodynamics), as such data are limited. Moreover, global mycotoxin regulations currently focus on major toxins, and are based on toxicity of individual mycotoxins, and have not considered potential additive or synergistic toxicity of combined mycotoxins. Effort should also be made to develop predictive models capable of predicting contamination from wider spectrum of mycotoxins and including climate change scenarios. Moreover, there is continuous need for development of novel detection and decontamination strategies for effective mycotoxin risk management. Mycotoxin risk management should be an integration of efforts that include risk assessment, establishment of regulatory options, as well as decontamination mitigation methods.

## Data Availability

Not applicable.

## Abbreviations

AA: Adsorbing agents
ADE: Apparent digestible energy
AF: Aflatoxins
AFB_1_: Aflatoxin B_1_
AFBO: Exo-AFB_1_-8,9-epoxide
BA: Biotransforming agents
BB: Brush border
CP: Crude protein
CYP: Cytochromes P450 enzymes
DM: Dry matter
DON: Deoxynivalenol
EFSA: European Food Safety Authority
FB_1_: Fumonisin B_1_
FUM: Fumonisins
GE: Gross energy
GST: Gutathione–S transferases
H/D: Ratio of villus height to crypt depth
IEC: Intestinal epithelial cells
Isc: Short-circuit current
LOAEL: Lowest-observed-effect-level
MDA: Mycotoxin-detoxifying agents
NA: Not applicable
NS: Not significantly affected
NOEL: No-observed-effect-level
OM: Organic matter
OT: Ochratoxins
OTA: Ochratoxin A
SGLT1: Sodium-dependent glucose co-transporter
TRC: Trichothecenes
UGT: UDP-glucuronosyltransferases
VFA: Volatile fatty acids
YCW: Yeast cell wall
ZEN: Zearalenone
